# The Relationality of Ecological Emotions: An Interdisciplinary Critique of Individual Resilience as Psychology’s Response to the Climate Crisis

**DOI:** 10.3389/fpsyg.2022.823620

**Published:** 2022-04-27

**Authors:** Weronika Kałwak, Vanessa Weihgold

**Affiliations:** ^1^Institute of Psychology, Jagiellonian University, Kraków, Poland; ^2^International Centre for Ethics in the Sciences and Humanities (IZEW), Eberhard Karls Universität, Tübingen, Germany; ^3^Centre Gilles Gaston Granger (CGGG), Aix-Marseille Université, Aix-en-Provence, France

**Keywords:** ecological emotions, relationality, resilience, climate crisis, climate change, climate emotions

## Abstract

An increasing number of academic papers, newspaper articles, and other media representations from all over the world recently bring climate change’s impact on mental health into focus. Commonly summarized under the terms of climate or ecological emotions, these reports talk about distress, anxiety, trauma, grief, or depression in relation to environmental decline and anticipated climate crisis. While the majority of psychology and mental health literature thus far presents preliminary conceptual analysis and calls for empirical research, some explanations of ecological emotions are already offered. They mainly draw from psychoanalysis and depth existential and humanistic psychology, as well as social psychology and address the relationship between ecological emotions and individual engagement in climate action. While these studies suggest building on individual resilience if concerned by ecological emotions, we argue that this only addresses their acute symptoms and not the (chronic) social causes. Based upon our literature research, we show that in an individualistic society such as the (neo-)liberal ones, feelings of individual responsibility are fostered, and this also applies to climate activism.

## Introduction

This paper presents results of an ongoing discussion between a philosopher and a psychologist—an ethicist and a mental health researcher—who are concerned with ecological emotions. The core of the discussion is the critique of philosophical and sociopolitical silent assumptions made in the current debate on ecological emotions in the discipline of psychology. The focus of the critique is the neoliberal idea of resilience that governs the process of knowledge generation and the official communication on the psychological aspect of the climate crisis and its influence on people’s mental health and wellbeing. It is beyond question that the current debate, and its core idea of resilience, influences the professional mental health education and support offered to individuals and communities touched by ecological emotions. Psychology of climate change is an emerging field that strives to meet an increasing need for mental health interventions addressing the climate change psychological impact (Doherty, 2018). There must be a space for an in-depth and critical reflection with respect to theoretical foundations of the field and resulting psychological practice, in order to ensure we adequately recognize specifics of mental health issues in the context of climate change (e.g., their strong interrelatedness with personal values and attitudes, as well as with the issues of politics and ethics). An important concern is to prevent mental health knowledge and interventions from legitimizing and producing climate injustice, or from consolidating unsustainable sociopolitical status quo. Psychology of climate change would then benefit from adopting critical and community psychology arguments against a iatrogenic individualization inherent to traditional psychology (Parker, 2007; Trickett, 2009; Kagan et al., 2022). We are aware that overcoming the ingrained individualistic assumptions requires a reform of research methodology and underlying conceptual structure, in the direction of participatory and collective transformative methodologies suggested by critical and community psychologies (Riemer and Reich, 2011; Lykes and Hershberg, 2012; Adams, 2021). While the latter show concern with climate change and do address its impact on mental health collectively, we still feel that Indigenous communities go a step further and thereby may help to overcome a conceptual impasse through counternarratives inspiring new ways of thinking and designing interventions (Wilson, 2008; Kimmerer, 2013; Cutter-Mackenzie-Knowles et al., 2020). Thus, we argue for a reconsideration of the psychological discourse that creates an ideal of the resilient individual who can adapt to the burden of the climate crisis. We propose a counternarrative of relationality that, in our view, may contribute to a more sustainable and just society, when adopted in mental health research and practice. Our aim here is to critically discuss the individualistic way in which ecological emotions are conceptualized in academic literature from the field of psychology and mental health studies. The emerging academic field of psychology of climate change, and the official message sent by international psychological associations, draw from the notion of resilience (Clayton et al., 2017; Doherty, 2018).

The arguments presented here reflect a broad and interdisciplinary critique of resilience being the focus of global policymaking in the face of various crises. The critique pertains also to framing adaptation to the climate crisis in terms of resilience in academic literature and in “grey” literature of national and international policymaking. In these contexts, the requirement of resilience is ascribed to communities, local socioeconomic systems, and natural environments in particular places of the world, at the background of the assumed directions of global adaptation to climate change. The assumed direction is usually constituted by the attempt to maintain global economic growth as the regulatory rule, despite the climate crisis and resources’ shortages worldwide. Therefore, the cost and burden of adaptation are situated locally (often in localizations of less power and resources) to prevent decrease in economic growth globally (Bottrell, 2009; Cannon and Müller-Mahn, 2010; MacKinnon and Derickson, 2013; McEvoy et al., 2013; Chapman et al., 2018; Doherty, 2018).

In our view, the very similar pattern may be identified in psychology of climate change that utilizes the concept of psychological resilience for building the understanding of ecological emotions—in at least three different domains: psychology of behavior change, psychology of mental health and wellbeing, and psychology of activism (Clayton and Manning, 2018). In general, employing psychological explanation in terms of knowledge-based global management of adapting to and mitigating climate change and encouraging massive psychological research in this field may be subjected to criticism. The reason for the criticism is underlying individualization and responsibilization of individuals against the global and systemic threat of the climate crisis. Discussion on resilience-based interventions in social work calls for bringing back the central place to social issues in professional understanding of mental health (especially in underprivileged populations that are susceptible to climate change impacts in the first place). We believe that adversity of official psychological discourses which individualize and psychologize socioeconomic and political processes should be broadly recognized and prevented in the emerging field of psychology of climate change (Nightingale and Cromby, 2001; Bottrell, 2009; Park, 2013). To sum up, in discussing psychological issue of ecological emotions through the lens of critical theory (Honneth, 2011; Rosa, 2018)—thus establishing a dialogue between psychology and philosophy—we attempt to understand emotional wellbeing in the individualist society which is facing the climate crisis. Another inspiration may come from the field of feminist psychotherapy that recognizes entanglement of our emotional suffering and the oppression of state and culture. It then claims the personal to be political and works for emancipation and raising political awareness through the mental health practice (Enns, 2011).

Generally speaking, the term *ecological emotions* address the emotional impact of environmental degradation. The term is frequently used in respect to emotional experience of people concerned with or touched by the consequences of global climate change (in this context, it is used interchangeably with *climate emotions*). However, it refers also to the emotional impact of environmental degradation of other origins, for example, brought about by mining [as in the classical research of Glenn Albrecht who has coined the term of *solastalgia* (Albrecht, 2005)] or pollution and contamination of water [as in the work on environmental melancholia of Lertzman (2015)]. Their common feature is that they all emerge in response to anthropogenic environmental degradation, so they open an experiential possibility for feelings of responsibility, mobilization, guilt, or shame (these emotions will be discussed further). It seems to reinforce an inclination inherent in the discipline of psychology to discursive responsibilization of individuals. In academic and popular debates, the emotional impact tends to be addressed with psychopathological terminology, especially in the context of global climate change (for example, *eco-anxiety*, *environmental grief*, and *climate depression*).

This impact, however, differentiates into two phenomena that are highly dependent on the regional contexts in which they occur. Thus, the first phenomenon is constituted by direct and indirect emotional and health consequences experienced by people who suffer from extreme weather events and environmental degradation caused by climate change (for example, from wildfires or sea-ice loss; Callaway et al., 1999; Cunsolo and Ellis, 2018). In these instances, we are dealing with a long wave increase in the deterioration in mental health of populations inhabiting the degraded regions, for example, with a high prevalence of suicide attempts among Australian farmers who experience recurring droughts (Bourque and Cunsolo, 2014). The other phenomenon seems to emerge in the localizations and populations that have not been yet so unequivocally touched by climate change-related extreme weather events and environmental degradation. In this context, the emotional impact is linked to the climate change awareness and environmental concern, which frequently co-exist with the anticipation of future destructive consequences of the climate crisis (Hayes et al., 2018). Additionally, it is often related to the involvement in various pro-environmental actions, including climate activism. In our paper, we focus primarily on this second phenomenon, so by ecological emotions, we mean what people feel and how they respond when emotionally touched by the global climate crisis and by the vision of possible future climate catastrophe. Importantly, regardless which of the two phenomena one has in mind, the impact of the climate crisis is not limited to how the condition of natural environment directly influences health and wellbeing. Just the opposite, socioeconomic, demographic, and political consequences of global climate change are the focus in ecological emotions.

First, we give more background information on ecological emotions. Second, we investigate resilience as a personal disposition addressed to build upon by psychological intervention and individualism as the underlying social structure that seems to establish this direction in conceptualizing and designing interventions. We address ecological emotions as social and political phenomenon. Thus, we attempt to show that the individualistic approach, and accompanying “psychologisation” (Parker, 2007) and “medicalization” of emotional responses to the climate crisis, is problematic both from the ethical-philosophical point of view, and from the perspective of mental health and wellbeing. Finally, we argue that an idea of relationality, inspired by feminist theories and indigenous philosophies (Butler, 2001; Wilson, 2008; Kimmerer, 2013), may serve as a counternarrative for this individualism. We believe that relationality as a foundation of our knowledge and action in the context of the climate crisis would contribute to sustainability and at the same time would support emotional wellbeing of communities threatened by the climate crisis.

Being from Europe, our perspective naturally is one from the Global North. This paper addresses the issue of ecological emotions in the Global North, because we want to point to a social problem we see in the industrialized neoliberal societies, we live in. We are well aware of the fact that people in various regions of the Global South are deeply touched by climate change and its emotional and health impacts. As well as of the interlinks and entanglements between the particular shapes the climate crisis takes on Global South and Global North. The call to study these issues in a contextualized way to prevent colonization of knowledge by Global North’s perspective is answered, among other researchers, by Verónica Iniguez-Gallardo and her colleagues in Ecuador (Martiskainen et al., 2020; Iniguez-Gallardo et al., 2021; Ogunbode et al., 2021).

## Ecological Emotions

There is a growing interest in ecological emotions not only in academic research but also in the broader public that shows in the number of articles published on the subject and that are not limited to research journals but also expand to newspapers, magazines, and digital media (Burger, 2013; Clayton and Manning, 2018; Haynes, 2019; Nugent, 2019; Wang et al., 2019; Cianconi et al., 2020; Vince, 2020).

Despite the fact that the words “ecological” and “climate” are contemporarily used in the first place to address the realms of a natural environment, their Greek etymology and deeper meaning are also linked to the sense of community, to the social or political climate, i.e., the affect that is created within and by a group of people. The term ecological is referring to the Greek concepts of *oikos* for “home” (including the larger family and all servants that lived there) and *logos* for “discourse.” As such it means “discourse about home and family.” Climate (gr. *klima*) on the other hand does not only refer to the long-term weather but also to the social or political climate, i.e., the affect that is created within and by a group of people. Arlie Hochschild, who exposed the link between human emotions, social life, and moral beliefs, offers a definition of emotion that we draw on.

Emotion, I suggest, is a biologically given sense and our most important one. Like other senses—hearing, touch, and smell—it is a means by which we know about our relation to the world, and it is therefore crucial for the survival of human beings in group life. Emotion is unique among the senses, however, because it is related not only to an orientation toward *action* but also to an orientation toward *cognition* (Hochschild, 2012).

The importance of group life is a focus of Hochschild’s definition, and this is also, in our view, the core point in both nature and etiology of ecological emotions. Both levels of meaning of the term *ecological*: the natural environment and the social aspect are then important for our argument, since they are strongly interconnected. As a result, we define ecological emotions as a sense that effectively informs us on the changing climate of our natural and social environment. The norm of rationality, that is still widespread in the neoliberal societies of the Global North, creates conventional restrictions on experiencing and expressing emotions (Boler, 1999). Emotions, and especially negative and painful emotions, are then declined as a possible resource for adequate and effective response to the climate crisis. Social and discursive norms of underestimating the role of emotion in understanding own natural and social environments may result in blocking own adequate recognition and action in the face of climate change by individuals and communities (Cvetkovich, 2007; Gould, 2010; Staiger et al., 2010). In the literature on ecological emotions, it is often argued that one might seldom find as *rational feelings* in the context of climate crisis. On the one hand, this discursive normalization of ecological emotions is based on the fact that there is not much doubt on the seriousness of the threat and the responsibility for the situation (Weintrobe, 2013; Marks et al., 2021). On the other hand, the neoliberal rational norm remains untouched, since this is the rationality what justifies and allows for an approval of emotional response. What is more, this is funded on simultaneous pathologization of climate denialism, which is presented no longer as a moral stance, but as psychological abnormality instead. Research on ecological emotions then should include a claim for a *new rationality* that does not exclude emotionality as an adequate regulatory power for experiencing and acting upon the climate crisis (Bladow et al., 2018; González-Hidalgo and Zografos, 2020).

Another inspiration here is then the understanding of affect and emotion offered by feminist theorists’ work on public feelings (Cvetkovich, 2007; Pile, 2010; Staiger et al., 2010). Affects and emotions are considered as having potential, as a source of drive for action, including political action or protest. And at the same time, they are open and subject to influence (e.g., to blocking their political potential by individualizing and pathologizing them). They may be then shaped in various ways, due to social, cultural, and political reasons and interests, including influencing the modes and directions of political action or protest that is emotionally fueled. Emotions may be also co-constructed by a discourse (for example, by the public discourse of academic psychology that is legitimized to produce an official body of knowledge on ecological emotions and on mental health). Therefore, the very fact of ecological emotions theorized as private inner feelings of an individual, as personal, intimate affairs—or as individually determined psychological abnormality in case of using medicalized terms of eco-anxiety or climate depression to address them—make them public and political issue. What is more, their openness to being shaped through discourses and their situatedness in the historical context of the urgent global climate crisis in neoliberal world allow to suspect that making sense of them as private and individual inner feelings is politically driven.

This section has given a general definition of ecological emotions and the theoretical background, we built upon. We have argued that ecological emotions are a sense that informs us about concern with the changing climate of our natural and social environment. The next part of this paper will discuss the recommended treatment of building individual resilience on a background of climate emotions as a social phenomenon.

## Individual Treatment for the Social Phenomenon of Ecological Emotions

The “Mental Health and our Changing Climate” report that has been issued by the *American Psychological Association* in 2017 addresses ecological emotions as a phenomenon of individual mental health and wellbeing. It claims as: “On an individual level, resilience is built internally and externally through strategies, such as coping and self-regulation, and community social support networks” (Clayton et al., 2017). The suggestions for intervention given to support resilience mainly focus on the individuals in working on their belief in self-efficacy, fostering their optimism, cultivating their coping strategies, finding a source of meaning, getting prepared for possible disasters, and connecting to the place they live. On the social side, the resilience should be strengthened by supporting a social network of an individual and a connection with the family and culture of origin. Resilience is first and foremost presented as an individual disposition that is preventive to negative emotionality and to deterioration of mental health and wellbeing, linked to perceptions of climate change.^1^ This individualistic understanding is also widespread in the media and ingrained in the everyday language that is used to address the emotional impact of climate change. One who is concerned with the climate crisis and touched by ecological emotions seems then to be suggested to build on own resilience to individually adapt to the emotional burden, both by environmental communication and by professional expertise that is produced to inform various forms of psychological education and intervention. We argue that this only addresses the acute symptoms and not the (chronic) social causes of ecological emotions. Based on our literature research, we reflect on the possible consequences this individualistic narrative of climate emotions may have as: for mental health and wellbeing, as well as for the ethical dimension of actions taken to mitigate climate change and to promote sustainability.

There are several points of critique based on the above suggestions for intervention some of them have been made in another context (Weihgold, 2021). First, in the industrialized culture of the Global North, feeling emotionally touched by climate change might create an inner conflict with one’s lived values based on productivity and traditional rationality. It might be true also for the family, the community, and the culture a person has grown into—experiencing and expressing environmental concern often is a source of interpersonal conflicts (Budziszewska and Kałwak, 2022). Therefore, intentions and attempts to find a new social network of people with similar values and ecological worldview are observed and may be supported in tailored interventions. Second, concerning the suggestions that are exclusively directed at the change that is happening intra-individually, we argue that most of them are addressing the emotional response in itself (what is more, a response that is constructed as undesired and often pathologized through psychological discourse) and not the emotion’s trigger, i.e., climate change. Self-efficacy, optimism, coping strategies, and meaning might help the individual person to learn to live with her feelings. But, first, it may indeed constitute a response unreflective to the seriousness of the threat and, second, it does not help solve the problem of climate change. In the pessimistic scenario, it may lead to accepting and adapting to the adversity instead of willing to work for a transformation, and consequently to consolidation and legitimization of adverse status quo (Bottrell, 2009; MacKinnon and Derickson, 2013). As in neoliberal societies, the system is understood as natural thus unchangeable. So, when the system cannot adapt, the citizen has to and therefore neoliberalism benefits from the concept of resilience (MacKinnon and Derickson, 2013). Third, preparing people (and communities) for possible disasters is necessary but still it remains addressing the symptoms.

Hartmut Rosa and Axel Honneth criticized (Honneth, 2011; Rosa, 2018) neoliberal societies in the Global North for their focus on individual rights and freedom that entails social pathologies (to use Honneth’s term) and a loss of the feeling of community. Neoliberal discourses of competitiveness promoting economic growth (that underpin the concept of resilience) create the space where every one person is responsible for succeeding in life (Ehrenberg, 1998). This is why, as Alain Ehrenberg sees it, there is a growing number of depressions in people who are not able to respond to this responsibility and then, they feel inadequate. At the same time, state regulations have been cut back since the 1990s not only in the economic sphere but in general (Honneth, 2011). This is the reason why, in our opinion, the notion of ecological emotions, apparently addressing an intrapersonal dimension of human psychological function, in fact has strong social and political underpinnings.

One of the most cited ecological emotions—eco-anxiety—is defined in the literature as a “chronic fear of environmental doom” (Clayton et al., 2017). However, in the majority of popular, educational, and media messages, as well as in the interviews conducted on subjective experience of people self-identifying with the label of eco-anxiety and climate depression, not a fear but a sense of an individual, personal responsibility and feelings of guilt and shame come to the fore. They frequently occur with various expressions of depressive resignation and suicidal ideation. This is especially so when particular lifestyles and consumer behaviors (e.g., individual reduction of carbon footprint) are co-constructed by these messages as necessary means to mitigate climate change, and some of them may be unaffordable due to socioeconomic status and family structure. This may be interpreted as a response to the experience of inner conflict between the feeling of individual responsibility, the urgency, and the enormous scale of the task that is impossible to fulfill by any individual.

Although the purpose of strengthening the individual resilience is to help people to cope with the threat of climate change, it does not address these social factors. Therefore, we argue that interventions provided to those who are touched by ecological emotions must also address the social context, social feelings, and their causes. As has been recently shown by Elizabeth Marks and colleagues in a quantitative study on eco-anxiety in 10 different countries of the world, young people not only feel anxious about their future but also feel betrayed by the inaction of political leaders (Marks et al., 2021). Already in 2006, Kari Norgaard reported on the fact that people feel helpless with regard to the immensity of the problem and the fact that states do not reach agreements on action (Norgaard, 2006). These findings show, as we have stated in the beginning, that there is an important social and political part in ecological emotions. Feelings of betrayal and helplessness signal that the social environment does not support sustainable lifestyles and leave climate action to the individuals.

As we already mentioned, in our current societies (in the Global North), there is a focus on individuality, responsibilizing people for the success of their life stories. In this line of thought, the logical consequence for treating ecological emotions is to address them with a focus on resilience, i.e., strengthening the coping abilities of the affected individuals so that they adapt to the stressful experience and can continue with own life tasks. But, as Ehrenberg (1998) already accented in his analysis on depression, the concentration on individuality is taken as a responsibility for a successful life. The depression then would be a reaction to the pressure one feels in succeeding in life and the impression of not being capable of it. This individual responsibility can also be found in resilience. Building the culture of resilience is an example of neoliberalism’s way of social mobilizing, both to pursue individuals’ engagement in global productivity, and involvement in climate action in case of individuals who are environmentally concerned. This is to say that in neoliberal states, the individual must pursue her values while state and economy pursue theirs (Joseph, 2013; MacKinnon and Derickson, 2013).

The inaction of states, official administrations, and big industries concerning climate change exemplify this responsibilizing and mobilizing of the individual citizens. Current climate action movements like Fridays for Future, or Extinction Rebellion, are organized from the bottom up and rooted in the feeling of need for a social change oriented toward sustainability. These analyses are in accordance with Foucault’s critique that the state has a right to sacrifice its citizens since they are only what it takes care of (Foucault, 1988) what makes them replaceable.

The argument for integrating the social context has already been made by community psychology. In the face of climate change, their focus is to build a community resilience through empowering practices like community gardening. But even though this is an important point and Oktavat and Zautra integrate Earth in their definition of community, their research on urban gardening does only highlight the question of resilience, that is recovery and sustained wellbeing of impacted systems. (Okvat and Zautra, 2011) Without question, community gardening is a practice that can help foster community and individual wellbeing through activities that mitigate climate change, but it does not change the underlying way of thinking and assumptions that should be transformed in the field of psychology.

From an ethical perspective, there are two problems with neoliberal thinking. Not only, the point that citizens are seen as mere resources for the needs of a state or economy, but also the fact that the individual responsibility for change is reinforcing the reasons for depression and anxiety. This is to say that an individual cannot tackle a global problem that concerns the whole of humanity. Therefore, the feeling of not succeeding and, as a result, that of depression (Ehrenberg, 1998) and the feeling of anxiety (being alone facing a huge threat) are reinforced. Therefore, as Budziszewska and Jonsson have shown, climate action can help address ecological emotions (Budziszewska and Jonsson, 2021).

Feeling emotionally touched by climate change thus is even reinforced by the experience of being left alone by political and economic leaders, when feelings of helplessness and isolation arise (Weintrobe, 2013; Pihkala, 2020). The individual responsibility and self-reliance in facing the climate crisis thus are also a key influence (MacKinnon and Derickson, 2013). A focus on collective climate action (frequently supported in popular and educational advice on how to deal with ecological emotions) shall overcome this individual isolation. In the context of activism, feelings of responsibility are constructed as value-laden in a way to encourage activism. At the same time, burnout and depression hence are believed to be frequent among activists. Through the call for adaptation and resilience, the responsibility for dealing with the emotional burden of climate action to secure energetic and emotional resources necessary to maintain their activism is also ascribed to the individuals. As in Ehrenberg’s analysis succeeding one’s life (thus, also succeeding in one’s activism), which includes one’s emotional wellbeing, is an important task for everyone. Having to stand up for failing this task because of burnout or depression is adding to the burden of not feeling well.

As a result, this discursive entanglement of individualistically understood psychological aspects of ecological emotions and of ethical and moral realms seems to impact emotional wellbeing of those who are concerned and committed to climate action. This is another argument for a reflection and reconsideration of the individualistic discursive tools that are used in psychology to address the phenomenon of climate emotions. There is a potential conflict between psychological expertise built in individualistic terms (as well as powerful position psychology has to influence or even define the social discourse), and psychological praxis as concerned with supporting and protecting people and communities in their mental health and wellbeing (Nightingale and Cromby, 2001). Researchers must reflect on this privileged and difficult position (Parker, 2007). As we intended to present, the current social discourse of resilience seems to position people’s wellbeing as instrumental to other aims and interests. It seems to be made through shifting responsibility for the mitigation of climate change from the state to individual citizens. First, it is done through discursive promotion of engagement in environmental activism and of individual change of behavior toward environmentally conscious lifestyle, both fueled by feelings of guilt and shame. Second, through building a sense of individual responsibility for maintaining these individual actions in time, despite the lack of individually noticeable success and actual chances for an individual to succeed. Resilience, that is constructed as an individual disposition partially determined by intraindividual biological and psychological mechanisms, is meant here to prevent from burnout (Wu et al., 2013; Davenport, 2017; Doherty, 2018). This is obviously the interest of the state that is trying to protect its traditional way of being dependent on the neoliberal idea of productivity, through austerity and other ways of dysregulation (MacKinnon and Derickson, 2013).

In the tradition of Foucault scientific theory is considered to be the most important influence on the social structures. Once a theory has gained the status of mainstream (by being spread by renowned international institutions like APA), it is followed by a majority. Psychology focusing primarily on the affective, emotional, and motivational deterministic mechanisms that are being situated inside the individual, and on the private subjective experiences of a person is sustaining the idea of neoliberalism as being the natural and unchangeable order. When generating theory on ecological emotions, it should provide insights into the broader sociopolitical and metatheoretical contexts. Psychology as a discipline in general has been criticized for an inadequate and adverse individualistic approach to human beings and human health. The internal self-reflection within the discipline, originating from critical, community, and feminist psychology, brings light to hidden legitimization and consolidation of sociopolitical status quo through “psychologization” of social norms and “naturalization” of the state, which are oppressive to people and to communities, thus should be subjected to change (Parker, 2007; MacKinnon and Derickson, 2013).

To sum up this part, addressing ecological emotions means being aware of their social and cultural factors, including ethical reflection, especially when various forms of psychological intervention are considered. The current focus on individual resilience has been criticized for not addressing the causing triggers of ecological emotions. An explanation for this recommendation has been identified in the general orientation of psychology toward the individual that takes the natural and social environment as a background of minor importance. But we argue that the individualization of ecological emotions—and binding these emotions with the sense of commitment to climate action and sustainable behavior—discursively sustains the shift of responsibility for mitigation of climate change toward individuals, while the necessary action should be taken by the state through systemic changes and policymaking. A necessary modification of these adverse assumptions made in the psychology of climate change seems difficult, since psychological expertise penetrates social discourses and everyday ways of making sense of, expressing, and coping with emotional suffering related to environmental crisis. Thus, transformative action should be undertaken not only with respect to psychological education and intervention, but also through adopting participatory methodologies of knowledge production, in order to collectively construct an expertise that prevents the faults discussed here. In the spirit of Berlin (1997), we propose to search for a pluralistic counternarrative. One that is not value-free but conscious of the sources of injustice and open for different values. While such counternarratives to the concept of resilience as resistance, resourcefulness, or vulnerability reduction are offered (Bottrell, 2009; Cannon and Müller-Mahn, 2010; MacKinnon and Derickson, 2013), we think we have found an example of this different discourse in the Indigenist concept of relationality, we will now turn to.

## Relational Counternarrative and How This Could Help Framing Ecological Emotions

Shawn Wilson defines relationality as “Rather than viewing ourselves as being *in* relationship with other people or things, we *are* the relationships that we hold and are part of.” (Wilson, 2008); thus, relationality also means to always be accountable in Indigenous Communities to the other members of the group. As opposed to Western societies, no one will stay anonymous, because references are always made to the experiences and the life of a person who witnessed. This opposition to anonymity seems to be in line with what Foucault critiques when talking about the invisible power structures of social discourse (Foucault, 2004). Only the difference lays in the fact of accountability to the discourse: while those in power in Western societies secretly control the narrative (for example, through the proxy which are the institutions legitimized to generate knowledge), in Indigenous societies they must be open and take responsibility for the good of the relationship. This is what we try to do in this paper by addressing the social discourse and official academic discourse of ecological emotions that is founded on the notion of resilience as one that should be transformed—in line with relationality, to ensure both the sustainability and just society.

As Judith Butler argues, relationality is the grounding for all forms of ethical judgments. Prior to judging the other, one must be in relation with her (Butler, 2001). As social beings, humans are depending on a group. To take part in, one means respecting the group’s norms. Therefore, the relations define the group. But if the group norms are built—as described above—on individualism, the concept is becoming difficult. As Cutter-Mackenzie-Knowles and colleagues remark as: neoliberal ideology, with its’ focus on individualism and exclusion has suppressed such interconnected ways of being and knowing (Cutter-Mackenzie-Knowles et al., 2020). Even though, as Ehrenberg lets us know, growing up in a society of individuals does not mean that one must become a monad that only interacts with other monads on economic markets or through legal contracts (Ehrenberg, 1998), we still see a tendency to this as we have mentioned above.

In considering ecological emotions, building a relation with others who have similar experiences or engaging oneself in a group of activists can create a ground for lifting the pressure (Pike, 2017). Therefore, for example, deep psychologist Sally Gillespie in her work with people affected by ecological emotions focuses on group therapy, and critical community psychologists suggest designing interventions based on the systemic approach that address whole communities instead of their individual members (Trickett, 2009; Kagan et al., 2022). “Individualism weakens the fabric of social connections, breeds loneliness and falsifies ecological realities” (Gillespie, 2020). In Gillespie’s view, people need to hear more personal stories and less statistics to respond to the challenges we face and to not feel alone. This is in line with relationality: Humans need relationships for their emotional wellbeing, be it to realize that there are others who feel the same or be it to find peer examples.

To address the problem of guilt, Gillespie recommends to reframe Climate Change as systemic problem and not to focus on the responsibility of individuals or groups. Another important point is that we also have to tackle the narrative of the lonely hero, if we want to seriously treat ecological emotions.

No one person can save the planet or ourselves from climate catastrophes, unlike in the movies. It is important to keep this in mind when committing to climate action. Both because the sense of urgency and pressure to act is high, and because the archetype of the warrior/hero is so dominant in contemporary cultures (Gillespie, 2020).

In our view, sharing experiences in a group creates a feeling of interconnectedness and gives examples for action to address the causes of ecological emotions. This does counter the neoliberal narrative of performant individuals.

But relationality is not only about solidarity and support. As a counternarrative to resilience, it also means caring for those who are directly touched, and thus vulnerable—not to alienate them through responsibilization and a constraint to adapt. For Indigenous philosophers, it is grounded in another worldview:

In contrast to dominant Western society’s tendency to view the natural world as a commodity, property, or a “resource,” Indigenous understandings are based on regarding the Earth as alive and imbued with spirit. In this view, a reciprocal set of duties and responsibilities between humans and the rest of the natural world exists such that, assuming these obligations are consistently met, relations between human and non-human entities are maintained in a healthy balance (McGregor et al., 2020).

As Robin Wall Kimmerer explains, relationships are created through gifts (Kimmerer, 2013). To maintain the balance, McGregor and colleagues are talking about, a gift is never free. It creates a bond between the giver and the receiver, and the latter must give something back if she does not want to cut this bond. In the Indigenous worldview, the Earth is also constantly giving (food, water, shelter…). Therefore, humans as receivers have the duty to care for the Earth in return (sowing seeds, keeping the water clean…). As this web of relations includes the whole Earth with all her beings, there are more rules to be respected. The most important is to never take all so that plants or animals will not be extinguished and so that other beings still can live by them (Kimmerer, 2013). This is the healthy balance McGregor and colleagues are talking about and it is going beyond Butler’s account of grounding ethical judgements.

When talking about relationships, the Indigenist worldview does not only think of other human beings but this rather includes the more-than-human, the land or country, the whole cosmos, and also ideas. Being the relationships we are part of, we are also part of or, in the words of Meira Baindur: “embodied in the functions of being related to the processes of natural resource degradation, by being an efficient cause.” (Baindur, 2015). This relational worldview comes with an ethical implication (and not only a judgment, as Butler suggested), that is that we need to care for these relations and thus care for everything.

But if we look at the people affected by ecological emotions based on relationality, and ask the question, who is keeping up the balance of giving and taking, the diagnosis for our social environment in the Global North would probably be catastrophic because people who are affected by these emotions feel that they are giving more than they receive. Creating a climate for change, as Susan Moser and Lisa Dilling invites us to do (Moser and Dilling, 2007), and addressing ecological emotions, in our opinion, therefore also means to address the underlying social discourse of individual responsibility.

## Discussion

This paper has argued that the theoretical treatment for ecological emotions that is emerging in psychology, that builds on the concept of individual resilience, is only addressing the acute symptoms of the phenomenon, and not the underlying social and environmental causes that trigger the emotions. First, we have given a general definition of ecological emotions as a sense informing on the changing climate of our natural and social environment and then, we have turned to looking into the treatment suggestion of individual resilience and the underlying social structure of ecological emotions. In the last part, we have suggested a relational counternarrative that could build our social being on interconnectedness and thus address the symptoms of ecological emotions we have been discussing.

Leaving the beaten paths of current psychological research with its focus on individual resilience that is inscribed into a generalized focus on the individual in neoliberal society seems to be what the current climate crisis is calling for. Thus, analyzing the emotional impact of this crisis on this background helps adding different perspectives, as we have seen with the relational worldview. This way, this paper contributes to a deeper understanding of the social implications of psychology in general and ecological emotions in specific. Nevertheless, this paper, with its sole theoretical and analytical focus, has its limitations. Research about ecological emotions conducted by Indigenous scholars seems to be completely lacking but could contribute to testing the counternarrative we have proposed. When it comes to the psychological practice, on the other hand, we wanted to contribute to a deeper reflection on the treatment, affected people will receive.

It was not the aim of our paper to call for an appropriation of Indigenous philosophy, but to learn from how human being can be perceived differently. Generally speaking the way, we are doing research is co-constructing the way we make sense of the world. If our assumptions are based on a relational perspective, it opens our eyes to different results. One consequence of this can be a relational focus in education, as proposed by Cutter-Mackenzie-Knowles et al. (2020), who are using relationality as a pedagogical concept for relating to Earth through walking, touching, and artistically engaging with nature. This could also be applied to psychological intervention in a way that “empowerment” is no more focused on just one individual or one group (thereby creating and in and out perspective), but taking the ecological thought of the interdependent network of the cosmos seriously.

## Author Contributions

All authors listed have made a substantial, direct, and intellectual contribution to the work and approved it for publication.

## Funding

The authors received a contribution to the publication fees by Tübingen University, the IZEW; for that moment we did not receive funding for the publication from Jagiellonian University, however we will apply for it soon and let the editors know as soon as there is a decision on that.

## Conflict of Interest

The authors declare that the research was conducted in the absence of any commercial or financial relationships that could be construed as a potential conflict of interest.

## Publisher’s Note

All claims expressed in this article are solely those of the authors and do not necessarily represent those of their affiliated organizations, or those of the publisher, the editors and the reviewers. Any product that may be evaluated in this article, or claim that may be made by its manufacturer, is not guaranteed or endorsed by the publisher.

## Footnotes

^1^There is a chapter on community resilience that suggests to foster access to health infrastructure, to train people in disaster management, and to reduce disparities and facilitating social cohesion among others. In our view, all of these are helpful but will not change underlying ways of thinking and approaching the world. As will become clear in the following, social cohesion can also be perceived as a burden, depending on the prevalent values.
